# The role of identity in parental support for physical activity and healthy eating among overweight and obese children

**DOI:** 10.1080/21642850.2020.1750959

**Published:** 2020-04-27

**Authors:** Alexander Lithopoulos, Sam Liu, Ryan E. Rhodes, Patti-Jean Naylor

**Affiliations:** School of Exercise Science, Physical & Health Education, University of Victoria, Victoria, Canada

**Keywords:** Physical activity, healthy diet, attitude, identity, parent support

## Abstract

**Introduction:** Parental support behaviors are established predictors of children's physical activity and healthy eating. However, little is known about predictors of these parental support behaviors. Identity (i.e. a component of the self-concept) has been hypothesized to be an influential construct that may be associated with a variety of behavioral antecedents and behavior itself. Specifically, research suggests healthy eating or physical activity parental support affective attitude may predict parental identity, and that identity may predict support behavior directly and indirectly through support self-regulation (e.g. planning). Thus, this study expands on past literature by exploring these antecedents and outcomes of parental identity in the context of parental support for physical activity and healthy eating among overweight and obese children.

**Methods:** Using a cross-sectional survey design, 83 parents (61.4% female) with at least one overweight or obese child completed measures assessing parental support affective attitude, parental identity, support self-regulation, and actual support behaviors. Path analysis was conducted to examine model fit and hypothesized relationships between variables for eating and physical activity separately.

**Results:** For both behavioral domains, the model fit was good. Parental support affective attitude predicted parental identity, parental identity predicted support self-regulation, and support self-regulation predicted support behavior. Further, for both behaviors, support self-regulation mediated the relationship between parental identity and support behavior. Finally, parental identity also directly predicted support behavior for physical activity but not eating.

**Conclusions:** The results demonstrate the importance of identity in parental support behaviors. These results also show that fostering enjoyable experiences for parents while supporting their children may strengthen their supportive identities.

Childhood obesity is one of the most common pediatric health problems and has been linked to multiple physiological and psychosocial problems such as high blood pressure and lower quality of life (Canning, Courage, & Frizzell, 2004; Maximova, Kuhle, Davidson, Fung, & Veugelers, 2013; Schwimmer, Burwinkle, & Varni, 2003). Without intervention, overweight children will likely continue to be overweight during adolescence and adulthood (Canning et al., 2004; Twig et al., 2016). If current trends continue, by 2040, up to 70% of adults aged 40 years will be either overweight or obese (Twig et al., 2016). Lifestyle behavioral interventions are the principal intervention approach for helping children maintain a healthy weight (Flynn et al., 2006; Kohl et al., 2012; Sacher et al., 2010; Tyson & Frank, 2018; Wang et al., 2013). Consistent moderate to vigorous physical activity (i.e. activity causing harder breathing and sweating; Godin & Shephard, 1985) and a diet composed of sufficient amounts of fruits and vegetables are important behaviors for preventing and managing childhood obesity and ensuring optimal health and development (Poitras et al., 2016; Morenga, & Montez, & M, 2017; Wang et al., 2014). Therefore, there is a need to study the determinants of these two health behaviors to provide some guidance regarding future intervention targets (Rothman, 2004).

Family-based lifestyle behavioral interventions may be more effective at helping children maintain a healthy weight if consistent parental support behaviors are present. Parental support behaviors for a healthy lifestyle are established key predictors of child physical activity and healthy eating (Scaglioni et al., 2018; Yao & Rhodes, 2015). Support has been conceptualized as responsiveness (i.e. giving encouragement) and providing structure (i.e. provide an optimal physical environment) and those parents that provide both to their children may be most likely to succeed at supporting healthy lifestyle behaviors (Davison, Li, Baskin, Cox, & Affuso, 2011). Thus, interventions increasing the regularity of these forms of parental support should in turn promote child health.

Knowledge regarding predictors of parental support behaviors in the physical activity and healthy eating domains (especially for overweight and obese children) is limited. However, in terms of physical activity, Rhodes et al. (2019a) recently demonstrated that parental identity toward supporting one's child to do regular moderate to vigorous physical activity better predicted parental support than all of the other psychological variables tested (support affective attitude, instrumental attitude, perceived capability, perceived opportunity, planning, habit). Identity can be defined as a component of a hierarchically organized self-concept made up of various roles (Markus, 1977; Stryker & Burke, 2000). According to identity theory, identities act as personal standards that are compared to current behavior. Any discrepancies between the two should create negative affect which would then motivate action to close the gap between identity and the present situation (Burke, 2006). Thus, parents who hold a parental physical activity identity (i.e. ‘It is my duty to be physically active and to ensure my child is consistently active’) and a parental healthy eating identity (i.e. ‘It is my job to ensure our family is consistently eating fruits and vegetables’) should be more likely to provide support (e.g. take child to a place where they can be physically active, buy fruits and vegetables) when they recognize a discrepancy between current behavior (e.g. being sedentary together, eating unhealthy together) and the identities. In terms of healthy eating, Strachan and Brawley (2009) demonstrated that strength of a healthy eater identity (i.e. ‘I am someone who eats healthy’) does predict healthy eating. Therefore, based on past literature, it is our belief that parental healthy eating or physical activity identity may be a potential target for interventions to increase parental support.

It is important to understand the antecedents of parental healthy eating or physical activity identity to know how to modify it. A review of identity in the physical activity domain found that commitment, self-efficacy, affective judgements, identified/integrated regulation (i.e. valuing the behavior and believing it is congruent with the self), and social activation (e.g. experiencing feelings of belonging) all reliably predicted identity (Rhodes, Kaushal, & Quinlan, 2016a). Affective judgements or attitude, which are emotional feelings about the behavior based on prior experiences and expectations (e.g. ‘The behaviour is pleasant’; Fishbein & Ajzen, 2010), may be particularly important. Affective attitude generally has a large-sized correlation with behavioral intentions, a medium-sized correlation with behavior (McEachan et al., 2016), and experimental studies in the physical activity domain show it is modifiable through intervention (Rhodes, Gray, & Husband, 2019b). The physical activity self-definition model (Kendzierski & Morganstein, 2009) also suggests that enjoyment predicts identity because enjoyment increases commitment to maintain the identity, and self-determination theory (Ryan & Deci, 2000) predicts that people will continue to do a behavior if they believe the behavior is enjoyable and congruent with the self. It therefore seems probable that affective attitude about parental support would predict parental identity for healthy eating or physical activity.

Identity may exert its effects on behavior both directly and indirectly (Caldwell et al., 2018; Kahneman, 2011; Rhodes, 2017). Direct effects may occur when the process is relatively automatic, reflexive, and beyond conscious awareness. There is empirical support for the idea that identity directly effects behavior for both healthy eating and physical activity (Carfora, Caso, & Conner, 2016; Rhodes et al., 2016a). A direct effect may occur when parental support behaviors occur immediately after the activation of the parental identity for healthy eating or physical activity. Regarding healthy eating, for example, a parent may be prompted to prepare a vegetable snack for one's child. Indirect effects may occur when the process is relatively slow, reflective, and within conscious awareness. Mediators of the relationship between parental identity and parental support behavior for healthy eating or physical activity are therefore important to identify because they explain how parental identity leads to parental support when we are consciously aware of what we are doing. Rhodes et al. (2016a) found that identity has reliably predicted self-regulatory behaviors such as goal setting, planning, and self-monitoring regarding the progress one is making on the behavior. These parental support self-regulation processes may then be mediators and there is some evidence supporting this idea. For example, Strachan, Brawley, Spink, Sweet, and Perras (2015) showed that self-regulation self-efficacy (i.e. confidence to self-regulate) was a mediator in the relationship between physical activity identity and exercise behavior, and Carraro and Gaudreau (2010) found planning to be a mediator. Rhodes et al. (2019a) and Rhodes et al. (2016b) also both showed parental physical activity support self-regulation was correlated with support behavior. These empirical findings are also consistent with identity theory. That is, the discrepancies between current behavior and the identity should motivate self-regulatory action to close the gap between identity and behavior (Stryker & Burke, 2000). Parental identity (e.g. for physical activity) may then cause parents to employ self-regulation strategies (e.g. plan to drive one's child to a place to play a sport) to ensure they provide support (e.g. actually drive one's child to a place to play a sport) to their children.

The purpose of this study was to examine the purported antecedents and outcomes of parental identity noted above in the context of parental support for physical activity and healthy eating among overweight and obese children. No research has yet examined these particular theoretical paths in one model for either behavior. It was hypothesized that affective attitude regarding parental support would predict parental identity, identity would predict parental self-regulation to support one's child, and self-regulation would then predict parental support for both child physical activity and healthy eating. Further, it was hypothesized that parental identity would also directly predict parental support, and support self-regulation would mediate the relationship between parental identity and parental support (see Figure 1).

**Figure 1.**
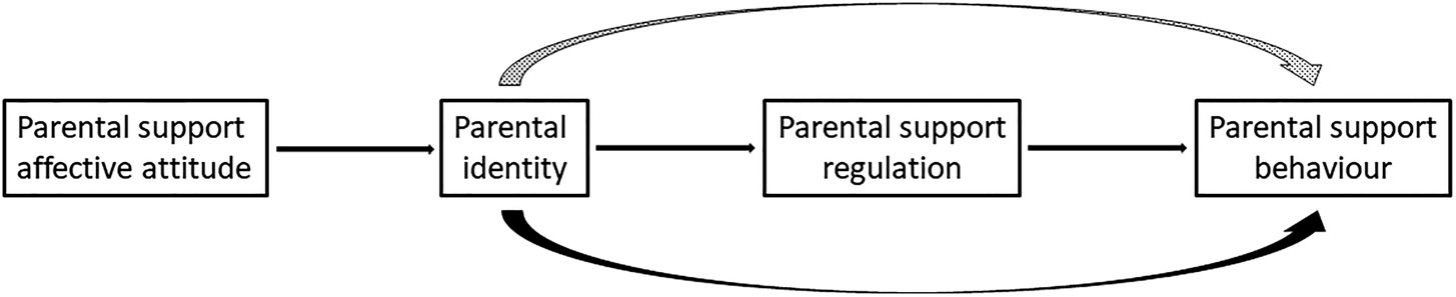
Hypothesized model. The dotted curved arrow represents an indirect effect and the solid curved arrow represents a direct effect controlling for the mediator.

## Method

### Design

This is a sub-study of a randomized waitlist-controlled trial (registered trial number: NCT03643341) that assessed the effectiveness of a 10-week interactive family-based lifestyle intervention for children 8–12 years of age (BMI ≥ 85th percentile of age and sex). Baseline data were analyzed in this study. Study participants were recruited from British Columbia, Canada. Participants completed the baseline survey from October 2018 to March 2019. Ethical clearance was formally provided by a university's human subject research ethics board for the trial and participants provided informed consent.

### Participants

Eighty-three parents (61.4% female), representing 89 overweight or obese children (*M* age = 10.58, *SD* = 1.55), completed the measures using an online survey at the baseline phase of a family-oriented intervention to manage childhood obesity. All children recruited were 8–12 years of age with a BMI ≥ 85th percentile of age and sex. Please see Table 1 for available demographic information.

**Table 1.** Available demographic information.

Variable	Frequency (%)	*M* (*SD*)
Child age (in years)		10.58 (1.55)
Child gender		
Male	29 (34.9)	
Female	24 (28.9)	
Did not indicate	30 (36.1)	
Parent Gender		
Male	9 (10.8)	
Female	51 (61.4)	
Did not indicate	23 (27.7)	
Single parent status		
Yes	22 (26.5)	
No	56 (67.5)	
Did not indicate	5 (6.0)	
Child background		
Aboriginal	7 (8.4)	
White	34 (41.0)	
Chinese	1 (1.2)	
South Asian	10 (12.0)	
Black	2 (2.4)	
Southeast Asian	8 (9.6)	
Japanese	1 (1.2)	
Korean	2 (2.4)	
Mixed	12 (14.5)	
Did not indicate	6 (7.2)	
Household income before taxes		
Under $28,000	11 (13.3)	
$28,000 to $33,999	4 (4.8)	
$34,000 to $40,999	5 (6.0)	
$41,000 to $46,999	3 (3.6)	
$47,000 to $52,399	5 (6.0)	
$53,000 to $58,999	2 (2.4)	
$59,000 or higher	39 (47.0)	
Did not indicate	14 (16.8)	
Education for primary caregiver		
Finished grade 10	1 (1.2)	
High school	21 (25.3)	
Vocational school or apprenticeship	13 (15.7)	
Community college	16 (19.3)	
University certificate	6 (7.2)	
Undergraduate degree	9 (10.8)	
Above undergraduate degree	10 (12.0)	
Did not indicate	7 (8.4)	

### Measures

*Affective attitude for healthy eating and physical activity support.* Two items, adapted from Rhodes, Blanchard, and Matheson (2006), measured parental support affective attitude for eating (α = .50) and physical activity (α = .80). This scale has shown adequate reliability and discriminant, construct, and predictive validity (Rhodes et al., 2006). The first item for each behavior was scored on a scale that ranged from 1 (*extremely unenjoyable*) to 7 (*extremely enjoyable*). The items were ‘For me, regularly supporting my child's (healthy eating [e.g. increasing fruit and vegetable consumption and decreasing intake of sugary beverages]/moderate-to-vigorous physical activity [e.g. driving to practice, scheduling activities]) over the next two weeks would be:’ The second item for each behavior was scored on a scale that ranged from 1 (*extremely unpleasant*) to 7 (*extremely pleasant*). The items were ‘For me, regularly supporting my child's (healthy eating/moderate-to-vigorous physical activity [e.g. driving to practice, scheduling activities]) over the next two weeks would be:’ Because reliability for eating affective attitude was poor, we included only the first item in subsequent eating analyses (see Appendix).

*Self-regulation for healthy eating and physical activity support.* Four items, adapted from previous research (Rhodes et al., 2016b; Rhodes et al., 2016b; Sniehotta, Schwarzer, Scholz, & Schüz, 2005), measured parental support self-regulation for eating (α = .86) and physical activity (α = .89). Areas of self-regulation targeted were based on evidence indicating which self-regulation behavior change techniques are most effective at influencing healthy eating and physical activity (Golley, Hendrie, Slater, & Corsini, 2011; Michie, Abraham, Whittington, McAteer, & Gupta, 2009). The items assessed goal setting (for the behavior; item 1), review of behavioral goals (item 2), problem solving/coping planning (item 3), and action planning (item 4). The items, scored on a scale that ranged from 1 (*strongly disagree*) to 5 (*strongly agree*), were ‘I set short-term (daily or weekly) goals for how I could support my child's (healthy eating/leisure-time physical activity) behaviors last month,’ ‘If I did not reach (my goal/one of my goals) for supporting my child's (healthy eating/physical activity) last month, I analyzed what went wrong,’ ‘I made plans regarding what to do if something made it difficult to support my child's (healthy eating/physical activity) last month,’ and ‘I made regular plans concerning “when”, “where”, “how”, and “what” kind of support I could provide for my child's (eating behaviors and food choices/physical activity) last month.’

*Parental identity for healthy eating and physical activity.* Three items, adapted from the role identity subscale of the Exercise Identity Scale (Anderson & Cychosz, 1994; Wilson & Muon, 2008), measured parental identity for eating (current study α = .82) and physical activity (current study α = .88). Wilson and Muon (2008) found the scale had adequate reliability (α = .84) and construct, convergent, and predictive validity. The eating items were ‘I consider myself an individual who prepares healthy food and beverage choices,’ ‘When I describe myself to others, I usually include my commitment to eating healthy,’ and ‘Others see me as someone who regularly eats healthy.’ The physical activity items were ‘I consider myself an exerciser,’ ‘When I describe our family to others, I usually include something about our physical activities,’ and ‘Others see us as a family that is regularly active.’ Each item was scored on a scale that ranged from 1 (*strongly disagree*) to 5 (*strongly agree*).

*Parental support for healthy eating and physical activity.* Commensurate with the responsiveness and structure dimensions of parental support, participants were asked questions about encouragement and logistics support. Three items, adapted from previous research (Davison et al., 2011; Rhodes et al., 2016b), measured parental support for eating (α = .82) and physical activity (α = .80). Davison et al. (2011) found the physical activity scale to be reliable (α = .72) and valid (construct and convergent validity tested). The healthy eating items were study created. The eating items began with the stem ‘During a typical week how often have you or another family member of your household:’ The items, scored on a scale that ranged from 1 (*not at all*) to 4 (*every day*), were ‘Encouraged your child to eat more fruit,’ ‘Encouraged your child to eat more vegetables,’ and ‘Bought fruit or vegetables you know your child likes.’ The physical activity items, scored on a scale that ranged from 1 (*strongly disagree*) to 5 (*strongly agree*), were ‘I watch my child play sports or participate in other activities such as martial arts or dance,’ ‘I enroll my child in sports teams and clubs such as soccer, basketball, and dance,’ and ‘I take my child to places where he/she can be active.’

### Data analysis

Path analysis with a covariance matrix using robust maximum likelihood estimation was conducted to examine model fit and the relationships between variables for eating and physical activity separately (Schumacker & Lomax, 2010). Total (i.e. the independent variable predicting the dependent variable), direct (i.e. the independent variable predicting the dependent variable controlling for the intervening variable), and indirect (i.e. the total effect minus the direct effect) relationship effects were examined to test the study's hypotheses (Preacher & Hayes, 2004). For indirect effect tests for both healthy eating and physical activity, the independent variable is parental identity, the mediator is support self-regulation, and the dependent variable is parental support behavior. Analyses were conducted using LISREL version 10.10. Several criteria were used to judge model fit: (a) significance and direction of relationships between variables, (b) size of standardized residuals between variables (greater than 3.29 was considered unacceptable), and model fit indices. The following indices (accompanied with acceptable fit guidelines) were used to assess how well the model fit the data: χ^2^ (non-significant value), GFI (.90 or higher), SRMR (less than .05), RMSEA (.08 or lower), and NFI (.90 or higher; Schumacker & Lomax, 2010). Also, as per guidelines provided by Cohen (1988), small, medium, and large effect sizes were interpreted when *R*^2^ was greater than or equal to .02, .13, and .26, respectively. Based on the planned analyses, using G*Power, we calculated needing a sample size of 77 participants to detect a medium effect size (ƒ^2^ = .15; Cohen, 1988) at 80% power with α = .05 (Faul, Erdfelder, Lang, & Buchner, 2007). Thus, the final sample size was slightly larger than this estimate.

## Results

### Data screening and preparation

Using SPSS version 24, the data were first examined for outliers at the univariate level (Tabachnick & Fidell, 2012). Univariate outliers were values more than three times greater or less than the middle 50% (25th to 75th percentiles) of the distribution (Pallant, 2011). No outliers were discovered. Next, patterns in missing data were examined for each behavior separately. The data were missing completely at random for eating (Little's MCAR test: chi-square = 18.68, DF = 29, *p* = .93) and physical activity variables (Little's MCAR test: chi-square = 32.04, DF = 24, *p* = .13). Imputation was then conducted through the expectation maximization procedure using LISREL version 10.10 for each behavior separately. Finally, multivariate outliers, participants with a Cook's distance greater than 1 (Tabachnick & Fidell, 2012), were investigated for each behavior separately; however, none were discovered.

### Parental support for healthy eating

**Figure 2.**
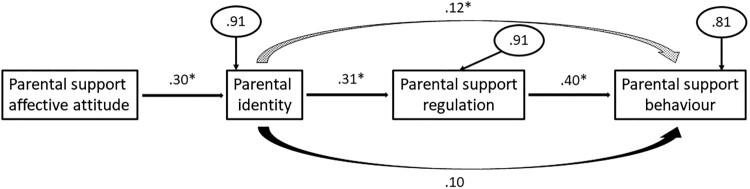
Standardized regression coefficients (and measurement errors in ellipses) for the healthy eating model. The dotted curved arrow represents an indirect effect and the solid curved arrow represents a direct effect controlling for the mediator. Statistically significant effects have an asterisk. Unstandardized indirect effect = .096, CIs = .012, .180.

The fit indices for this model are as follows: χ^2^ = 1.07 (df = 2), *p* = .59, GFI = .99, SRMR = .04, RMSEA = .00, and NFI = .97. There were no large standardized residuals and nearly all of the relationships between variables were also significant and in the positive direction (see Table 2 for the correlation matrix). Therefore, the available information suggest that this model fits the data well (see Figure 2). Parent support affective attitude had a small-sized association with parental identity (*β* = .30, *p* < .01, *R*^2^ = .09), parental identity had a small-sized association with support self-regulation (*β* = .31, *p* < .01, *R*^2^ = .09), and support self-regulation significantly predicted parental support (*β* = .40, *p* < .01). The direct effect between parental identity and parental support (i.e. controlling for support self-regulation) was not significant (*β* = .10, *p* = .38), and the variance explained in parental support from parental identity and support self-regulation was in the medium-sized range (*R*^2^ = .19). There was also a positive indirect effect between parental identity and parental support through support self-regulation (*β* = .12, *p* < .05; see Table 3 for means and standard deviations).

**Table 2.** Correlation matrices.

Variable	Affective attitude	Identity	Regulation	Support
Eating				
Affective attitude	1.00			
Identity	.21	1.00		
Regulation	.30	.31	1.00	
Support	.10	.43	.22	1.00
Physical activity				
Affective attitude	1.00			
Identity	.07	1.00		
Regulation	.25	.44	1.00	
Support	.24	.37	.41	1.00

**Table 3.** Means and standard deviations.

Variable (scale points)	*M* (*SD*)
Eating	
Affective attitude (1–7)	4.70 (1.73)
Identity (1–5)	3.27 (.86)
Regulation (1–5)	2.94 (.85)
Parental support (1–4)	3.08 (.68)
Physical activity	
Affective attitude (1–7)	5.42 (1.17)
Identity (1–5)	2.61 (1.04)
Regulation (1–5)	3.09 (.82)
Parental support (1–5)	3.67 (.99)

### Parental support for physical activity

**Figure 3.**
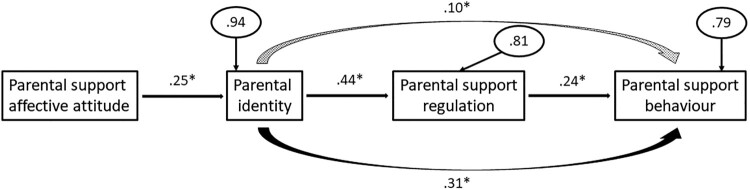
Standardized regression coefficients (and measurement errors in ellipses) for the physical activity model. The dotted curved arrow represents an indirect effect and the solid curved arrow represents a direct effect controlling for the mediator. Statistically significant effects have an asterisk. Unstandardized indirect effect = .124, CIs = .001, .247.

The fit indices for this model are as follows: χ^2^ = 2.12 (df = 2), *p* = .35, GFI = .99, SRMR = .04, RMSEA = .06, and NFI = .95. There were no large standardized residuals and all of the relationships between variables were also significant and in the positive direction (see Table 2 for the correlation matrix). Therefore, the available information suggest that this model fits the data well (see Figure 3). Parental support affective attitude had a small-sized association with parental identity (*β* = .25, *p* = .02, *R*^2^ = .06), parental identity had a medium-sized association with support self-regulation (*β* = .44, *p* < .001, *R*^2^ = .19), and support self-regulation significantly predicted parental support (*β* = .24, *p* = .03). The direct effect between parental identity and parental support (i.e. controlling for support self-regulation) was significant (*β* = .31, *p* < .01), and the variance explained in parental support from parental identity and support self-regulation was in the medium-sized range (*R*^2^ = .21). There was also a positive indirect effect between parental identity and parental support through support self-regulation (*β* = .10, *p* < .05; see Table 3 for means and standard deviations).

## Discussion

The purpose of this study was to examine antecedents and outcomes of parental identity in terms of physical activity and healthy eating among overweight and obese children. We postulated that affective attitude regarding parental support would predict parental identity, identity would predict parental self-regulation to support one's child, and self-regulation would then predict parental support for both child physical activity and healthy eating. Further, we hypothesized that parental identity would also directly predict parental support, and support self-regulation would mediate the relationship between parental identity and parental support. To our knowledge, this is the first study to examine the relationships between these variables in a single model among parents of children who are overweight or obese. Nearly all of the hypotheses were supported for both behaviors. Our results have important research and clinical implications as they highlight key factors that may lead to improvements in parental support behavior for their child to engage in regular physical activity and eat a healthy diet.

First, from a theoretical perspective, this study advances knowledge of the identity construct regarding parental support in the physical activity and healthy eating domains. According to identity theory (Burke, 2006; Stryker & Burke, 2000), discrepancies between a current self and one's identity should produce distress and motivate self-regulation and subsequent behavior change to reduce any discrepancies. In the current study, for both behavioral domains, support self-regulation mediated the relationship between parental identity and parental support. Stated another way, viewing oneself as a healthy eater or physically active parent motivated support self-regulatory behaviors (i.e. goal setting, self-reflection, planning) which then, in turn, motivated parental support behaviors (i.e. encouragement and logistical support). Thus, these findings are consistent with identity theory. Also, the pattern of findings are consistent with behavioral theories that propose reflective (indirect, mediated) and reflexive (direct) paths to behavior stemming from identity (Caldwell et al., 2018; Kahneman, 2011; Rhodes, 2017). Evidence of the latter was also observed because there was indeed a direct effect from parental identity to parental support for physical activity. The direct effect suggests that identity can also affect behavior in a manner not associated with self-regulation in certain situations. The lack of a direct effect for healthy eating may indicate that eating parental support requires self-regulation such as planning meals for one's family.

The results also indicated that support affective attitude predicted parental identity for health eating and physical activity. This effect has been consistently demonstrated in the physical activity domain (Rhodes et al., 2016a). Also, the physical activity self-definition model (Kendzierski & Morganstein, 2009) would predict the same effect, and self-determination theory (Ryan & Deci, 2000) suggests that individuals need to enjoy the behavior and believe the behavior is congruent with the self in order for behavioral maintenance to occur. Therefore, support affective attitude predicting parental identity is consistent with related theories. However, this effect had not yet been demonstrated in the parental support literature. This finding suggests that parents may require some sort of enjoyment during parental support activities for healthy eating or physical activity in order for the identity to develop and strengthen over time. Practically, or from a clinical perspective, this knowledge also provides guidance for intervention. It suggests that parental support affective attitude be targeted in interventions.

Affective attitude is modifiable (Rhodes et al., 2019b), so future research should investigate whether an experiment can influence parental identity through support affective attitude change. There are no published identity interventions in the physical activity domain beyond one feasibility trial (i.e. Husband, Wharf Higgins, & Rhodes, 2018) and only a handful exist in the healthy eating domain (e.g. Brouwer & Mosack, 2015; Carfora, Caso, & Conner, 2017). Regarding potential intervention targets, self-determination theory (Ryan & Deci, 2000) would suggest enhancing parent autonomy, relatedness, and competence in terms of support, and hedonic theory (e.g. Zajonc, 1980) would suggest making support behaviors more pleasurable. In terms of enhancing the pleasure of the experience during the provision of support, we know that people tend to prefer immediate rewards compared to delayed ones (Metcalfe & Mischel, 1999). Thus, interventions would need parents to think that providing support is worth it in the short-term. Perhaps the implementation of a reward system or conditioning parents to think of support as being enjoyable (e.g. through Pavlovian or evaluative conditioning) would positively influence parental support affective attitude for healthy eating or physical activity (de Ridder, Kroese, Evers, Adriaanse, & Gillebaart, 2017; Mantzari et al., 2015; Rhodes & Kates, 2015). Certainly, more research is needed to learn how support affective attitude can be manipulated to encourage the development of strong parental identities for healthy eating and physical activity.

A strength of this study is the use of previously validated questionnaires to measure support affective attitude, identity, self-regulation, and parental support behaviors for physical activity and diet. Additionally, this study focused on examining the support behaviors for a healthy lifestyle in a critical population group, parents of children who are overweight or obese. Family-focused behavioral weight management interventions are the principal intervention approach for achieving long-term weight control in children (Flynn et al., 2006; Kohl et al., 2012; Sacher et al., 2010; Tyson & Frank, 2018; Wang et al., 2013). Thus, understanding the factors contributing to health eating and physical activity parental support behavior is important. A limitation of the present study was the small sample size. Also, our sample did not have a diverse ethnicity and social-economic status background. Furthermore, our healthy eating measures focused on evaluating fruit and vegetable intake. Other components such as dietary fat intake were not measured. These factors limit the generalizability of our findings. Another limitation was the lack of longitudinal data over a lengthy period. Longitudinal data can be used to build more complex multivariate models with the ability to better examine both mediation and moderation effects to help inform mechanisms of change. Future study in this area is warranted. Finally, our healthy eating support affective attitude measure contained only one item which limited our understanding of its reliability.

## Conclusion

This study examined the predictors of parental support for physical activity and healthy eating among parents who have children who are overweight and obese. We found that that affective attitude about parental support predicts parental identity, identity predicts parental self-regulation to support their child, and self-regulation predicts parental support for both child eating and physical activity. Also, there were indirect and direct effects of parental identity to parental support. These results reinforce the importance of fostering enjoyable experiences for parents while supporting their children in order to strengthen their healthy eating and physical activity parental identities.

## Supplementary Material

Supplemental Material

## Appendix: Full Measures

*Affective Attitude*:

Healthy Eating:

For me, regularly supporting my child's healthy eating (e.g. increasing fruit and vegetable consumption and decreasing intake of sugary beverages) over the next two weeks would be:

**Table:**

Extremely unenjoyable	Quite unenjoyable	Slightly unenjoyable	Neutral	Slightly enjoyable	Quite enjoyable	Extremely enjoyable

For me, regularly supporting my child's healthy eating over the next two weeks would be:

**Table:**

Extremely unpleasant	Quite unpleasant	Slightly unpleasant	Neutral	Slightly pleasant	Quite pleasant	Extremely pleasant

Physical Activity:

For me, regularly supporting my child's moderate-to-vigorous physical activity (e.g. driving to practice, scheduling activities) over the next two weeks would be:

**Table:**

Extremely unenjoyable	Quite unenjoyable	Slightly unenjoyable	Neutral	Slightly enjoyable	Quite enjoyable	Extremely enjoyable

For me, regularly supporting my child's moderate-to-vigorous physical activity (e.g. driving to practice, scheduling activities) over the next two weeks would be:

**Table:**

Extremely unpleasant	Quite unpleasant	Slightly unpleasant	Neutral	Slightly pleasant	Quite pleasant	Extremely pleasant

*Self-Regulation*:

Healthy Eating:

I set short-term (daily or weekly) goals for how I could support my child's healthy eating behaviors last month.

**Table:**

Strongly disagree	Disagree	Neutral	Agree	Strongly agree

If I did not reach my goal for supporting my child's healthy eating last month, I analyzed what went wrong.

**Table:**

Strongly disagree	Disagree	Neutral	Agree	Strongly agree

I made plans regarding what to do if something made it difficult to support my child's healthy eating last month.

**Table:**

Strongly disagree	Disagree	Neutral	Agree	Strongly agree

I made regular plans concerning ‘when,’ ‘where,’ ‘how,’ and ‘what’ kind of support I could provide for my child's eating behaviors and food choices last month.

**Table:**

Strongly disagree	Disagree	Neutral	Agree	Strongly agree

Physical Activity:

I set short-term (daily or weekly) goals for how I could support my child's leisure-time physical activity last month.

**Table:**

Strongly disagree	Disagree	Neutral	Agree	Strongly agree

I made regular plans concerning ‘when,’ ‘where,’ ‘how,’ and ‘what’ kind of support I could provide for my child's physical activity last month.

**Table:**

Strongly disagree	Disagree	Neutral	Agree	Strongly agree

I made plans regarding what to do if something made it difficult to support my child's physical activity last month.

**Table:**

Strongly disagree	Disagree	Neutral	Agree	Strongly agree

If I did not reach one of my goals for supporting my child's physical activity last month, I analyzed what went wrong.

**Table:**

Strongly disagree	Disagree	Neutral	Agree	Strongly agree

*Identity*:

Healthy Eating:

I consider myself an individual who prepares healthy food and beverage choices.

**Table:**

Strongly disagree	Disagree	Neutral	Agree	Strongly agree

When I describe myself to others, I usually include my commitment to eating healthy.

**Table:**

Strongly disagree	Disagree	Neutral	Agree	Strongly agree

Others see me as someone who regularly eats healthy.

**Table:**

Strongly disagree	Disagree	Neutral	Agree	Strongly agree

Physical Activity:

I consider myself an exerciser.

**Table:**

Strongly disagree	Disagree	Neutral	Agree	Strongly agree

When I describe our family to others, I usually include something about our physical activities.

**Table:**

Strongly disagree	Disagree	Neutral	Agree	Strongly agree

Others see us as a family that is regularly active.

**Table:**

Strongly disagree	Disagree	Neutral	Agree	Strongly agree

*Parental Support:*

Healthy Eating:

During a typical week how often have you or another family member of your household:

Encouraged your child to eat more fruit.

**Table:**

Not at all	Sometimes	Almost every day	Every day

Encouraged your child to eat more vegetables.

**Table:**

Not at all	Sometimes	Almost every day	Every day

Bought fruit or vegetables you know your child likes.

**Table:**

Not at all	Sometimes	Almost every day	Every day

Physical Activity:

I watch my child play sports or participate in other activities such as martial arts or dance.

**Table:**

Strongly disagree	Disagree	Neutral	Agree	Strongly agree

I enroll my child in sports teams and clubs such as soccer, basketball, and dance.

**Table:**

Strongly disagree	Disagree	Neutral	Agree	Strongly agree

I take my child to places where he/she can be active.

**Table:**

Strongly disagree	Disagree	Neutral	Agree	Strongly agree
