# Supplementary material for: The role of identity in parental support for physical activity and healthy eating among overweight and obese children
Source: Health Psychol Behav Med. 2020 Apr 27;8(1):185–201. doi: 10.1080/21642850.2020.1750959 (PMC8114343; doi:10.1080/21642850.2020.1750959)
Supplement: Supplemental Material [file RHPB_A_1750959_SM9900.docx]

**Appendix A: Full Measures**

**Affective Attitude:**

Healthy Eating:

For me, regularly supporting my child’s healthy eating (e.g. increasing fruit and vegetable consumption and decreasing intake of sugary beverages) over the next two weeks would be:

| Extremely unenjoyable | Quite unenjoyable | Slightly unenjoyable | Neutral | Slightly enjoyable | Quite enjoyable | Extremely enjoyable |
| --- | --- | --- | --- | --- | --- | --- |

For me, regularly supporting my child’s healthy eating over the next two weeks would be:

| Extremely unpleasant | Quite unpleasant | Slightly unpleasant | Neutral | Slightly pleasant | Quite pleasant | Extremely pleasant |
| --- | --- | --- | --- | --- | --- | --- |

Physical Activity:

For me, regularly supporting my child’s moderate-to-vigorous physical activity (e.g. driving to practice, scheduling activities) over the next two weeks would be:

| Extremely unenjoyable | Quite unenjoyable | Slightly unenjoyable | Neutral | Slightly enjoyable | Quite enjoyable | Extremely enjoyable |
| --- | --- | --- | --- | --- | --- | --- |

For me, regularly supporting my child’s moderate-to-vigorous physical activity (e.g. driving to practice, scheduling activities) over the next two weeks would be:

| Extremely unpleasant | Quite unpleasant | Slightly unpleasant | Neutral | Slightly pleasant | Quite pleasant | Extremely pleasant |
| --- | --- | --- | --- | --- | --- | --- |

**Self-Regulation:**

Healthy Eating:

I set short-term (daily or weekly) goals for how I could support my child’s healthy eating behaviors last month.

| Strongly disagree | Disagree | Neutral | Agree | Strongly agree |
| --- | --- | --- | --- | --- |

If I did not reach my goal for supporting my child’s healthy eating last month, I analyzed what went wrong.

| Strongly disagree | Disagree | Neutral | Agree | Strongly agree |
| --- | --- | --- | --- | --- |

I made plans regarding what to do if something made it difficult to support my child’s healthy eating last month.

| Strongly disagree | Disagree | Neutral | Agree | Strongly agree |
| --- | --- | --- | --- | --- |

I made regular plans concerning “when”, “where”, “how”, and “what” kind of support I could provide for my child’s eating behaviors and food choices last month.

| Strongly disagree | Disagree | Neutral | Agree | Strongly agree |
| --- | --- | --- | --- | --- |

Physical Activity:

I set short-term (daily or weekly) goals for how I could support my child’s leisure-time physical activity last month.

| Strongly disagree | Disagree | Neutral | Agree | Strongly agree |
| --- | --- | --- | --- | --- |

I made regular plans concerning “when”, “where”, “how”, and “what” kind of support I could provide for my child’s physical activity last month.

| Strongly disagree | Disagree | Neutral | Agree | Strongly agree |
| --- | --- | --- | --- | --- |

I made plans regarding what to do if something made it difficult to support my child’s physical activity last month.

| Strongly disagree | Disagree | Neutral | Agree | Strongly agree |
| --- | --- | --- | --- | --- |

If I did not reach one of my goals for supporting my child’s physical activity last month, I analyzed what went wrong.

| Strongly disagree | Disagree | Neutral | Agree | Strongly agree |
| --- | --- | --- | --- | --- |

**Identity:**

Healthy Eating:

I consider myself an individual who prepares healthy food and beverage choices.

| Strongly disagree | Disagree | Neutral | Agree | Strongly agree |
| --- | --- | --- | --- | --- |

When I describe myself to others, I usually include my commitment to eating healthy.

| Strongly disagree | Disagree | Neutral | Agree | Strongly agree |
| --- | --- | --- | --- | --- |

Others see me as someone who regularly eats healthy.

| Strongly disagree | Disagree | Neutral | Agree | Strongly agree |
| --- | --- | --- | --- | --- |

Physical Activity:

I consider myself an exerciser.

| Strongly disagree | Disagree | Neutral | Agree | Strongly agree |
| --- | --- | --- | --- | --- |

When I describe our family to others, I usually include something about our physical activities.

| Strongly disagree | Disagree | Neutral | Agree | Strongly agree |
| --- | --- | --- | --- | --- |

Others see us as a family that is regularly active.

| Strongly disagree | Disagree | Neutral | Agree | Strongly agree |
| --- | --- | --- | --- | --- |

**Parental Support:**

Healthy Eating:

During a typical week how often have you or another family member of your household:

Encouraged your child to eat more fruit.

| Not at all | Sometimes | Almost every day | Every day |
| --- | --- | --- | --- |

Encouraged your child to eat more vegetables.

| Not at all | Sometimes | Almost every day | Every day |
| --- | --- | --- | --- |

Bought fruit or vegetables you know your child likes.

| Not at all | Sometimes | Almost every day | Every day |
| --- | --- | --- | --- |

Physical Activity:

I watch my child play sports or participate in other activities such as martial arts or dance.

| Strongly disagree | Disagree | Neutral | Agree | Strongly agree |
| --- | --- | --- | --- | --- |

I enroll my child in sports teams and clubs such as soccer, basketball, and dance.

| Strongly disagree | Disagree | Neutral | Agree | Strongly agree |
| --- | --- | --- | --- | --- |

I take my child to places where he/she can be active.

| Strongly disagree | Disagree | Neutral | Agree | Strongly agree |
| --- | --- | --- | --- | --- |
